# Case report: Personalized computational model guided ablation for left atrial flutter

**DOI:** 10.3389/fcvm.2022.893752

**Published:** 2022-09-15

**Authors:** Matthias Lange, Eugene Kwan, Derek J. Dosdall, Rob S. MacLeod, T. Jared Bunch, Ravi Ranjan

**Affiliations:** ^1^Nora Eccles Harrison Cardiovascular Research and Training Institute, The University of Utah, Salt Lake City, UT, United States; ^2^Biomedical Engineering, The University of Utah, Salt Lake City, UT, United States; ^3^Department of Surgery, Division of Cardiothoracic Surgery, University of Utah School of Medicine, Salt Lake City, UT, United States; ^4^Scientific Computing and Imaging Institute, The University of Utah, Salt Lake City, UT, United States; ^5^Department of Internal Medicine, Division of Cardiovascular Medicine, University of Utah School of Medicine, Salt Lake City, UT, United States

**Keywords:** atypical left atrial flutter, personalized computational model, predicting atrial flutter, ablation, prospective study

## Abstract

Atypical atrial flutter is seen post-ablation in patients, and it can be challenging to map. These flutters are typically set up around areas of scar in the left atrium. MRI can reliably identify left atrial scar. We propose a personalized computational model using patient specific scar information, to generate a monodomain model. In the model conductivities are adjusted for different tissue regions and flutter was induced with a premature pacing protocol. The model was tested prospectively in patients undergoing atypical flutter ablation. The simulation-predicted flutters were visualized and presented to clinicians. Validation of the computational model was motivated by recording from electroanatomical mapping. These personalized models successfully predicted clinically observed atypical flutter circuits and at times even better than invasive maps leading to flutter termination at isthmus sites predicted by the model.

## Introduction

Atypical left atrial flutter (ALAF) is an often-stable arrhythmia commonly seen in approximately 80% of the 20% of ablations for atrial fibrillation that results in atrial arrhythmias (1). The most promising treatment for ALAF is ablation, with reported acute success rates between 51% and 100% (2, 3). However, just one-year post-ablation, the recurrence is reported between 23% and 62% (2). The recurrence pathways in flutter are different from original pathways (3), meaning previously unobserved flutters are created. Thus, to prevent a recurrence, additional information besides the current activation pattern is required.

Computer simulation provides a powerful option to leverage patient-specific information and predict atrial arrhythmias (4, 5). ALAF is typically a macro-reentrant circuit around areas of scar that can be visualized using the Late-Gadolinium-Enhancement MRI (LGE-MRI). Attempts to use computer simulation for ALAF pathways (5) have been limited because they depended on invasive electro-anatomical mapping studies to inform the models rather than deriving them from non-invasive medical imaging alone.

We report here promising results from personalized computational models based entirely on information derived from LGE-MRI that can predict ALAF prospectively.

## Methods

### Patient selection

The study was approved by the Internal Review Board of The University of Utah. The inclusion criteria were to have at least one prior left-atrial ablation, planned ablation for ALAF, and a pre-procedural LGE-MRI.

### Geometric model generation

The left atrial LGE-MRI was obtained as previously described (6) and linearly interpolated to a resolution of 0.625 mm, 0.625 mm, and 0.625 mm. An affine registration was performed to align the magnetic resonance angiography (MRA) to LGE-MRI images. The left atrial wall and blood pool were segmented from the MRA using Corview (The University of Utah, Salt Lake City, United States). To segment the scarred regions, we set an intensity-based threshold according to the function


$$f\,\left(w\right)\to \left(w-\bar{B}\right)/s\,\left(B\right),$$

where, *w* is a pixel in the LGE-MRI, *B* is the set of all pixels in the blood pool with mean value $\bar{B}$ and sample standard deviation *s*(*B*). The value of *f* (*w*) served as a threshold for segmentation into the category of fibrosis (2 < *f* (*w*) < 3) or scar(*f* (*w*) ≥ 3).

From the wall segmentation, the surface model was extracted with the Iso2Mesh library (7). The endocardial surface was isolated and moved outward by 1.5 mm to approximate the epicardial surface. On both instances, the scar and fibrosis segmentations were projected.

The final step of creating a volumetric model of the geometry was to build a tetrahedral mesh using TetGen (WIAS, Berlin, Germany) based on the endocardial and epicardial surface models. To also include realistic myocardial fiber structure, we mapped the averaged myocardial fiber orientation from seven patients (8) to the new mesh. Finally, the mesh was optimized with openCARP (9) to have an edge length 400 to 900 μm.

### Computation simulation

We used the open-source package openCARP (9) to implement the monodomain model with membrane behavior described by the Courtemanche model of human atrial cells (10). The ionic conductivities were modified as listed in Table 1, which also lists the associated conduction velocities.

**TABLE 1:** Ionic conductivity multiplicators and conduction velocity.

Conductance	Symbol	Healthy	Fibrosis
Transit outward *K*^+^	g_*to*_	0.80	1.00
Maximal L-type inward *Ca*^2+^	g_*Ca,L*_	0.20	0.30
Inward rectifier *K*^+^	g_*K1*_	0.90	0.50
Maximal rapid delayed rectifier *K*^+^	g_*Kr*_	1.60	1.00
Fast inward *Na*^+^	g_*Na*_	1.00	0.80
Conduction velocity			
Longitudinal		0.95 m/s	0.89 m/s
Transversal		0.45 m/s	0.31 m/s

We carried out Virtual EP studies which tested 72 different combinations of pacing locations and intervals. A total of nine different pacing sites were simulated. At each site a train of eight pulses with a cycle length of 600 ms was delivered, followed by a single S2 beat. The S2 coupling interval started ranged from 250 ms to 180 ms in 10 ms intervals leading to eight different simulations for each pacing location. The simulation was run for a minimum of 700 ms after the last paced beat, and if tissue was still active at that point, the simulation was continued for a minimum of 1,700 ms to observe any arrhythmias. If ALAF was induced, we used the activation pattern to identify flutter circuits. Depending on the flutter circuit and scar distribution, potential ablation sites were identified.

### Clinical procedure

After the simulations were completed, all flutter circuits and virtual ablation results were presented to the electrophysiologist before the procedure. If the patient was in a flutter, the episode was mapped. Otherwise, a map was created in sinus rhythm and the pacing was conducted to induce flutter. For all the mapping studies CARTO (Biosense Webster, Irvine, CA, United States) mapping system was used. The pentaray catheter was used to make the maps and a few thousand points were collected for these maps as is routine for these studies. Ablation was done by using the Smarttouch ablation catheter. Both the activation map and the voltage map were visually compared to the scar segmentation and predicted flutter path. The simulation results together with the clinical findings were used by the electrophysiologist when ablating. During the electrophysiology study, an activation map of the flutter was made. Then the ablation sites were chosen based on the prediction of the simulation and the invasive map made during the electrophysiology study. If the two were congruent then the ablation was carried out at that site. If not, then the ablation was done first based on the map. If the flutter still persisted ablation sites predicted by simulation were ablated.

## Results

### Clinical results population

Four patients were recruited and underwent personalized computational model development and prediction of left atrial flutter circuits before the procedure. Maps from the first three cases showed a single stable flutter path which was also predicted by the computational model. As shown in Table 2, the computational model also predicted additional pathways that could sustain a flutter. In case four only two of the three clinically observed flutters were predicted by the model.

**TABLE 2:** Simulation and clinical summary.

Mapping study results	Case 1	Case 2	Case 3	Case 4	Mean ± Std
Age (years)	82	78	81	78	1.5 ± 1
Number of previous ablations	1	1	1	2	
Years since ablation	7	2	5	0.5	
Number of observed flutters	1	1	1	3	
Number of flutter targeted and terminated	1	1	1	3	1.5 ± 1
# of points	3720	2521	6200	1275	
Simulation results					
Number of predicted flutter	4	5	3	3	3.75 ± 0.96
Number of predicted flutters found	1	1	1	2	1.25 ± 0.5
Sensitivity	100%	100%	100%	66%	83%

**FIGURE 1:**
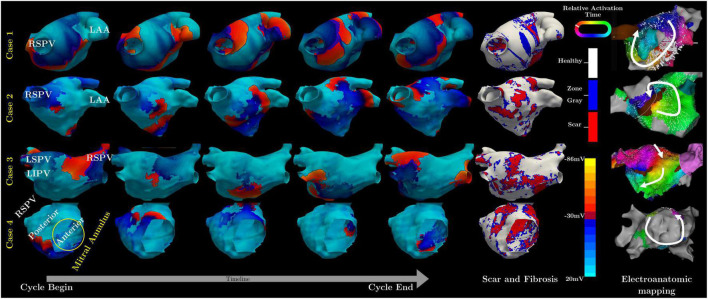
Summary of simulated and measured flutter circuits in four subjects. The left-hand panel contains a sequence of five simulated maps of transmembrane potential throughout a single re-entry cycle for each of the four cases. The sixth column shows the geometric model with scar and fibrosis, as obtained from LGE-MRI. The rightmost column contains the activation maps measured from each case.

Specifically, in case one the model predicted a flutter circuit around the right superior pulmonary vein (RSPV) (Figure 1), which matched the clinical observation. The critical circuit included a slow conduction region that, when ablated (between the RSPV and right interior pulmonary vein), terminated the flutter.

In the second case, the model showed reentry arising from an incomplete RSPV isolation line, leading to a figure of eight patterns (Figure 1). Mapping during sinus rhythm confirmed slow conduction in the posterior wall and the flutter circuit was induced on Isuprel and terminated with ablation.

**FIGURE 2:**
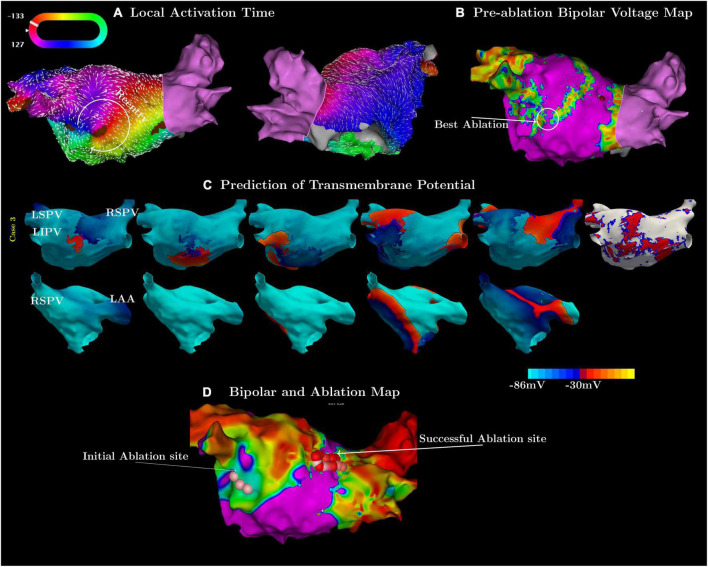
Examples in which the simulations were more accurate in predicting flutter circuits than high-density mapping. **(A)** Contains the pre-ablation activation map and **(B)** the associated bipolar voltage maps of the flutter. **(C)** Shows the simulated flutter with an isthmus on the posterior wall. **(D)** Shows a post-ablation map labeled with the initial (pink spheres), failed first ablation site, and the subsequent (red spheres), successful site of ablation.

In the third case, the simulation and clinical observations did not match at first (Figure 2). The measured activation map showed a slow conduction region inferior to the left interior pulmonary vein (LIPV) (Figures 2A,B). However, ablation there did not terminate, or even change the flutter cycle length or activation pattern. The simulation suggested a different circuit with a critical isthmus on the posterior wall of the LA (Figure 2C). Subsequent ablation of this region terminated the flutter successfully (Figure 2D).

In the last case, there was no stable circuit. The patient presented in sinus rhythm and attempts to induce flutter during the mapping procedure resulted in three non-sustained or self-terminating flutter circuits. One such circuit spread around the mitral valve and was also predicted from the simulation (Figure 1). A second circuit on the anterior wall in the form of a figure-of-eight pattern was predicted and found clinically. The third circuit found during mapping was a rotor on the posterior wall below the LIPV, which the simulations failed to confirm.

## Discussion

We present the development of new personalized computational models based entirely on LGE-MRI to determine anatomy and substrate information that prospectively predict left atrial flutter circuits, including the critical isthmus. In one example, the model prediction was even more accurate than the high-density activation map of the flutter. This was likely because of the extensive regions of scar presenting with very low voltage, making it difficult to correctly identify the local activation time, a common problem when dealing with ALAF.

Our model in general predicted more potential flutter circuits than those observed clinically. These additional circuits reveal themselves in response to multiple pacing sites and could be latent circuits that become clinically significant at a later time. The support for this interpretation comes from cases in patients who show new circuits after initial ALAF ablation. We will monitor the patients in this study for recurrence from such new circuits and compare them to those predicted by the simulations. It is also possible that some of these predicted additional circuits arose from inaccuracies in the scar determination or modeling of ionic currents. Despite these limitations, the results are very promising and underscore the potential benefits of exploring possible sustained circuits prospectively.

Scar in the left atrium is an essential part of ALAF and therefore it is essential for the personalized model of ALAF. We have shown for the first time in this setting that scar information is available from modern MRI with sufficient precision to enable accurate predictions of reentrant ALAF patterns.

## Data availability statement

The original contributions presented in this study are included in the article/supplementary material, further inquiries can be directed to the corresponding author.

## Ethics statement

The studies involving human participants were reviewed and approved by IRB at the University of Utah. The patients/participants provided their written informed consent to participate in this case study. Written informed consent was obtained from the participants for the publication of this case report and other publications based on data from this study.

## Author contributions

ML and RR contributed to the conception and design of the study and wrote sections of the manuscript. EK organized the database and got the clinical data. ML wrote the first draft of the manuscript. DD, RM, and TJB wrote sections of the manuscript. All authors contributed to the manuscript revision, read, and approved the submitted version.
